# Multiple attacks of transient monocular visual loss in a previously healthy man: a possible complication after COVID-19 vaccination?

**DOI:** 10.1186/s40942-022-00393-1

**Published:** 2022-06-20

**Authors:** Leonardo Provetti Cunha, Ângelo Atalla, José de Melo Costa-Neto, Luciana Virgínia Ferreira Costa-Cunha, Rony Carlos Preti, Leandro Cabral Zacharias, Mário Luiz Ribeiro Monteiro

**Affiliations:** 1 Department of Ophthalmology, Federal University of Juiz de Fora Medical School, Juiz de Fora, MG Brazil; 2 Department of Hematology, Federal University of Juiz de Fora Medical School, Juiz de Fora, MG Brazil; 3 Juiz de Fora Eye Hospital, Av. Rio Branco, 4051, Bom Pastor, Juiz de Fora, MG 36021-660 Brazil; 4 Department of Ophthalmology, University of São Paulo Medical School, São Paulo, Brazil

**Keywords:** Retinal vasospasm, Amaurosis fugax, Transient monocular visual loss, COVID-19 vaccine, Pfizer-BioNTech, Optical coherence tomography angiography

## Abstract

**Background:**

The present case aims to describe a previously healthy man who presented multiple attacks of transient monocular visual loss after Pfizer-BioNTech COVID-19 vaccination and to discuss the possible mechanisms related to occurrence of this condition.

**Case presentation:**

We report a case of multiple attacks of transient monocular visual loss in a previously healthy middle-aged man two weeks after Pfizer-BioNTech COVID-19 vaccination. TVL attacks were described as sudden and painless complete visual loss, lasting about one minute, followed by a full recovery. He presented several non-simultaneous attacks in both eyes, 16 in the right eye, and 2 in the left eye on the same day, fifteen days after receiving the second dose of the Pfizer-BioNTech COVID-19 vaccine. The brain’s magnetic resonance angiography, echocardiogram, and doppler ultrasound imaging of the carotid and vertebral arteries were non-revealing. The complete blood exam revealed a slightly elevated C-reactive protein test. We assessed fundus examination during the transient visual loss attack and revealed diffuse vascular narrowing for both arterial and venous branches, notably in the emergence of the optic disc in right eye. In addition, the circumpapillary optical coherence tomography angiography (OCTA) vessel density map was reduced. Oral verapamil hydrochloride 60 mg twice daily was initiated, and the attacks of transient visual loss improved after two days.

**Conclusions:**

To date, and the best of our knowledge, this is the first case report of multiple transient monocular visual loss attacks due to retinal vasospasm in a previously healthy middle-aged man documented by fundus retinography and OCTA. We discuss in this article the possible association of retinal vasospasm and Pfizer-BioNTech COVID-19 vaccination, probably related to vaccine-induced inflammation.

## Background

The COVID-19 pandemic, started in December 2019, led to sanitary, social, and economic impact at the global level, resulting in massive morbidity and mortality rates. Since then, a great search for treatments and vaccines against the virus began, with unprecedented efforts in the history of humankind. As a result, large-scale vaccination significantly impacted the number of hospitalizations and deaths and represents a successful strategy against the COVID-19 pandemic [1]. On the other hand, this mass vaccination brought many complications, some mild and transient, such as local pain, myalgia, fever, and weakness and, others potentially serious, such as myelitis, myocarditis, infarction, stroke, and thrombosis [2–4]. Many visual complications related to COVID-19 vaccination have been reported, such as diplopia, acute macular neuroretinopathy, central serous retinopathy, retinal vein thrombosis, papilledema, optic neuritis, uveitis, multiple evanescent white dot syndrome, Vogt-Koyanagi-Harada disease and Graves’ disease [5–9].

The present case aims to describe a previously healthy man who presented multiple attacks of transient monocular visual loss after Pfizer-BioNTech COVID-19 vaccination and to discuss the possible mechanisms related to occurrence of this condition.

## Case presentation

A 50 years-old previously healthy white man, with no migraine history, presented multiple episodes of monocular transient visual loss (TVL). TVL attacks were described as sudden and painless complete visual loss, lasting about one minute, followed by a full recovery without any prodromal signs or other visual or systemic symptoms. He described several non-simultaneous attacks in both eyes (OU), 16 in the right eye (OD) and 2 in the left eye on the same day. Fifteen days before, the patient received the second dose of the Pfizer-BioNTech COVID-19 vaccine, without any side effects, only a mild arm sore at the injection site. There is no previous history of COVID-19 infection, systemic arterial hypertension, or any other previous medical condition. In addition, the patient denied the use of any medication. He was referred to the emergence department, and the stroke fast track investigation protocol was proceeded. Neurological examination was unremarkable. Magnetic resonance angiography of the brain, echocardiogram, and doppler ultrasound imaging of the carotid and vertebral arteries were non-revealing. Complete blood count, Westergren erythrocyte sedimentation rate, D-dimer level, Antithrombin III, fibrinogen and protein C and S tests; serum electrophoresis, C3 and C4, anti-dsDNA, anti-Ro, anti-Sm, circulating immune complexes, indirect immunofluorescence for anti-nuclear antibodies on HEP-2 cells, anticardiolipine-lgM and IgG tests were normal. Serological tests for infectious diseases such as HIV, syphilis, toxoplasmosis, *Bartonella henselae*, cytomegalovirus, herpes simplex were negative. The C-reactive protein test was slightly elevated (8,3 mg/dl, reference value up to 6,0 mg/dl). The next day, the patient was referred for ophthalmic evaluation. The best-corrected visual acuity was 20/20 in OU. External eye examination, ocular motility, and anterior segment were normal. The pupils were equal in size, with no afferent pupillary defect. On the fundus exam, no emboli or other `abnormalities were found.

**Fig. 1:**
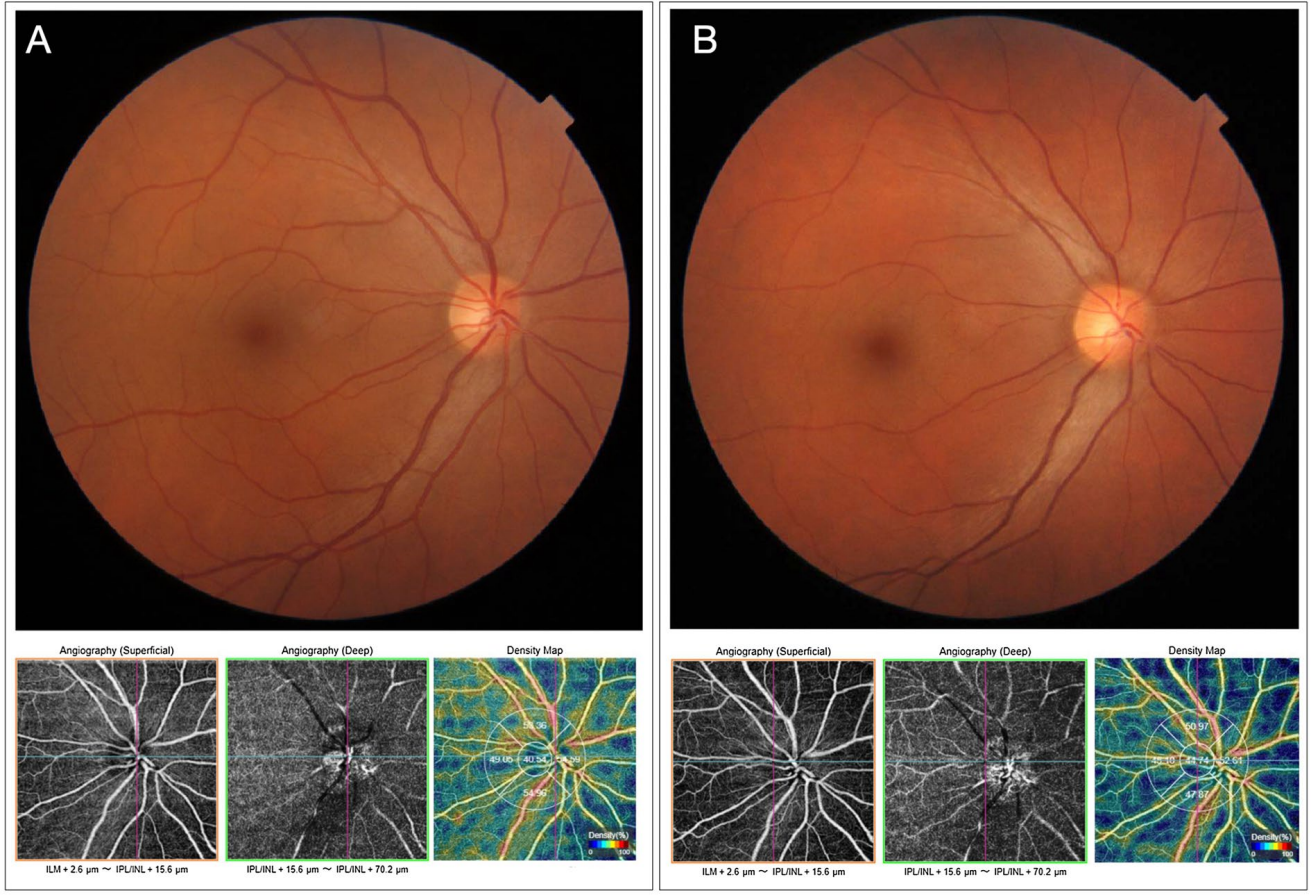
Fundus retinography (upper images) and optical coherence tomography angiography (OCTA) (bottom images) of the right eye. **A** Before de vasospasm attack. **B** During the vasospasm attack. Note in the fundus retinography (**B**), vasoconstriction and diffuse both arteriolar and venous narrowing. The OCTA en face images showed a marked reduction of both superficial and deep capillaries plexus, and the density map

Optical coherence tomography (OCT) and OCT angiography (OCTA) scanning of both optic disc and macular area were within normal limits. Automated visual field testing was also normal. However, the patient reported another episode of sudden visual loss in OD while in the waiting room. Fundus photography during the attack, revealed diffuse vascular narrowing for both arterial and venous branches, notably in the emergence of the optic disc. In addition, the circumpapillary OCTA vessel density map was reduced. Figure 1 shows both fundus and OCTA images before and during the attack. So, the retinal vasospasm diagnosis was made. Oral verapamil hydrochloride 60 mg twice daily was initiated, and the attacks of TVL disappeared after two days.

## Discussion and conclusions

Transient monocular visual loss, or amaurosis fugax, is caused by an abrupt and temporary reduction of blood flow to the optic disc, choroid, and retina [10]. There are several causes related to this condition, and thromboembolism, is one of the leading causes. However, extensive investigation ruled out any emboli source. Furthermore, fundus photography during the event clearly showed that retinal vasospasm was the mechanism in our case, as suggested in several previous studies [11, 12].

Ocular vasospasm in conditions where the inflammatory mechanism may play an important role had been well established, such as giant cell arteritis, systemic lupus erythematosus, infectious diseases, antiphospholipid antibody syndrome, Bechet’s disease, and rheumatoid arthritis [10, 13].

Most of these conditions can lead to vasospasm by increasing intravascular concentration of endothelin-1, a peptide produced and released by vascular endothelial cells. In addition, interleukin-1, a peptide involved in the inflammatory process, can increase the production of endothelin-1. Therefore, the combination of these events may justify the vasospasm secondary to an inflammatory process [10].

The occurrence of inflammatory events related to COVID-19 vaccination has already been described in previous studies, such as myocarditis, Guillain-Barré syndrome, transverse myelitis, neuromyelitis optica, optic neuritis, and others intraocular inflammatory processes [4–6, 14].

A recently published case reported a bilateral transient visual field defect experienced by a 42-year-old Thai ophthalmologist after the COVID-19 vaccination (CoronaVac, Sinovac Biotech Ltd). He presented left congruous hemianopia with respect to the vertical midline in the visual field test. The authors hypothesized that the possible mechanism was acute vasospasm of the posterior visual pathway, triggered by the CoronaVac vaccine [15]. As in our case, the authors did not find any other condition that could be associated with vasospasm.

In a previous report, Santovito and Pinna described a case of acute visual acuity and visual field loss after the second dose of the Pfizer-BioNTech COVID-19 vaccine [16]. In this case, the patient reported several visual and systemic symptoms suggestive of migraine, possibly triggered by COVID-19 vaccination. This report, like ours, reinforces the possibility of vasospasm events triggered after the Pfizer-BioNTech COVID-19 vaccine.Kindly check and confirm the 'Funding and Competing interests' statements are correctly processed.Done. It is correct. 

In accordance with vascular and inflammatory theory, other studies have reported several ocular inflammatory conditions related to COVID-19 vaccination. For example, Valenzuela et al. reported a case of acute macular neuroretinopathy immediately following the Pfizer-BioNTech COVID-19 vaccine administration [17]. Another article reported several inflammatory ocular events after COVID-19 vaccination, including the Oxford-AstraZeneca, ModernaTX, Janssen Johnson & Johnson, and Pfizer-BioNTech vaccines [18].

Although multiple attacks of transient visual loss due to retinal vasospasm possibly triggered by vaccine-related inflammatory events seem reasonable, we cannot assume that these events are directly related. It is possible that the retinal vasospasm could have occurred by chance or secondary to an underlying condition.

In conclusion, to date, and the best of our knowledge, this is the first case report of multiple transient monocular visual loss attacks due to retinal vasospasm after the Pfizer-BioNTech COVID-19 vaccination documented by fundus retinography and OCT angiography. The probably related mechanism was vaccine-induced inflammation. Although mass vaccination is considered an important tool in the fight against the COVID-19 pandemic, our case serves to emphasize that, we must be aware of its potential side effects to diagnose and treat appropriately.

## Data Availability

The datasets used and/or analyzed during the current study are available from the corresponding author on reasonable request.

## References

[1] Lopez Bernal J, Andrews N, Gower C, Robertson C, Stowe J, Tessier E (2021). Effectiveness of the Pfizer-BioNTech and Oxford-AstraZeneca vaccines on covid-19 related symptoms, hospital admissions, and mortality in older adults in England: test negative case-control study. BMJ.

[2] Patone M, Mei XW, Handunnetthi L, Dixon S, Zaccardi F, Shankar-Hari M (2021). Risks of myocarditis, pericarditis, and cardiac arrhythmias associated with COVID-19 vaccination or SARS-CoV-2 infection. Nat Med..

[3] Kim HW, Jenista ER, Wendell DC, Azevedo CF, Campbell MJ, Darty SN (2021). Patients with acute myocarditis following mRNA COVID-19 vaccination. JAMA Cardiol..

[4] Goss AL, Samudralwar RD, Das RR, Nath A (2021). ANA investigates: neurological complications of COVID-19 vaccines. Ann Neurol.

[5] Testi I, Brandão-de-Resende C, Agrawal R, Pavesio C, Group C-VOIES (2022). Ocular inflammatory events following COVID-19 vaccination: a multinational case series. J Ophthalmic Inflamm Infect.

[6] Ng XL, Betzler BK, Testi I, Ho SL, Tien M, Ngo WK (2021). Ocular adverse events after COVID-19 vaccination. Ocul Immunol Inflamm.

[7] Pichi F, Aljneibi S, Neri P, Hay S, Dackiw C, Ghazi NG (2021). Association of ocular adverse events with inactivated COVID-19 vaccination in patients in Abu Dhabi. JAMA Ophthalmol.

[8] Eleiwa TK, Gaier ED, Haseeb A, ElSheikh RH, Sallam AB, Elhusseiny AM (2021). Adverse Ocular Events following COVID-19 Vaccination. Inflamm Res.

[9] Alhumaid S, Al Mutair A, Al Alawi Z, Rabaan AA, Tirupathi R, Alomari MA (2021). Anaphylactic and nonanaphylactic reactions to SARS-CoV-2 vaccines: a systematic review and meta-analysis. Allergy Asthma Clin Immunol.

[10] Flammer J, Pache M, Resink T (2001). Vasospasm, its role in the pathogenesis of diseases with particular reference to the eye. Prog Retin Eye Res.

[11] Burger SK, Saul RF, Selhorst JB, Thurston SE (1991). Transient monocular blindness caused by vasospasm. N Engl J Med.

[12] Bernard GA, Bennett JL (1999). Vasospastic amaurosis fugax. Arch Ophthalmol.

[13] Phatak S, Jaison J, Soman M, Mohan A, Nair RU (2020). Retinal vasospastic phenomenon in a known case of systemic lupus erythematosus. Indian J Ophthalmol.

[14] Abbate A, Gavin J, Madanchi N, Kim C, Shah PR, Klein K (2021). Fulminant myocarditis and systemic hyperinflammation temporally associated with BNT162b2 mRNA COVID-19 vaccination in two patients. Int J Cardiol.

[15] Jumroendararasame C, Panyakorn S, Othong R, Jumroendararasame A, Srimanan W, Tipparut K (2021). Transient visual field loss after COVID-19 vaccination: experienced by ophthalmologist, case report. Am J Ophthalmol Case Rep.

[16] Santovito LS, Pinna G (2021). Acute reduction of visual acuity and visual field after Pfizer-BioNTech COVID-19 vaccine 2nd dose: a case report. Inflamm Res.

[17] Valenzuela DA, Groth S, Taubenslag KJ, Gangaputra S (2021). Acute macular neuroretinopathy following Pfizer-BioNTech COVID-19 vaccination. Am J Ophthalmol Case Rep.

[18] Bolletta E, Iannetta D, Mastrofilippo V, De Simone L, Gozzi F, Croci S (2021). Uveitis and other ocular complications following COVID-19 vaccination. J Clin Med..

